# Clinical and Radiographic Outcome of Non-Surgical Endodontic Treatment Using Calcium Silicate-Based Versus Resin-Based Sealers—A Systematic Review and Meta-Analysis of Clinical Studies

**DOI:** 10.3390/jfb13020038

**Published:** 2022-04-07

**Authors:** Viresh Chopra, Graham Davis, Aylin Baysan

**Affiliations:** 1 Department of Adult Restorative Dentistry, Oman Dental College, Centre for Oral Bioengineering, Queen Mary University, Mile End Rd. Bethnal Green, London E1 4NS, UK; 2 Centre for Oral Bioengineering, Institute of Dentistry, Faculty of Medicine and Dentistry, Queen Mary University of London, Mile End Rd. Bethnal Green, London E1 4NS, UK; g.r.davis@qmul.ac.uk (G.D.); a.baysan@qmul.ac.uk (A.B.)

**Keywords:** calcium-silicate-based sealers, bioceramic sealers, resin-based sealers, obturation, postoperative pain, extrusion

## Abstract

The aim of this paper is to systematically analyse the effect of calcium silicate-based sealers in comparison to resin-based sealers on clinical and radiographic outcomes of non-surgical endodontic treatment in permanent teeth. Methods: The study was conducted according to the guidelines of the Cochrane Handbook for Systematic Reviews of Interventions and Preferred Reporting Items for Systematic Review and Meta-Analyses (PRISMA) statement. The literature search was performed using PubMed/MEDLINE, Cochrane Central Register of Controlled Trials, Web of Science, DOAJ and OpenGrey with no language restrictions. Two reviewers critically assessed the studies for eligibility. Grading of Recommendations, Assessment, Development and Evaluations (GRADE) was carried out to assess the evidence. Meta-analysis of the pooled data with subgroups was carried out using the RevMan software (*p* < 0.05). Results: Results from the included studies showed that there were no significant differences between the groups in the 24 h post-obturation pain levels (mean difference (MD), −0.19, 95% CI = −0.43–0.06, *p* = 0.14, I^2^ = 0%), but at 48 h (MD, −0.35, 95% CI = −0.64–0.05, *p* = 0.02, I^2^ = 0%), a significant difference was observed in favour of calcium silicate sealers. Furthermore, there were no significant differences between the two sealers due to risk of onset or intensity of postoperative pain, need for analgesic and extrusion of the sealer. The heterogeneity assessed using Q test between the included studies was 97% (I^2^). Conclusions: Within the limitations of this review, the paper shows that calcium silicate-based sealers exhibited optimal performance with similar results to resin-based sealers in terms of average level of post-obturation pain, risk of onset and pain intensity at 24 and 48 h. The observations from the included studies are informative in the clinical evaluation of calcium silicate-based sealers and provide evidence for the conduction of well-designed, controlled randomised clinical trials for a period of at least four years in the future.

## 1. Introduction

Elimination of microorganisms is one of the prime requisites for the success of root canal treatment. Three-dimensional (3D) obturation of the root canal system plays a vital role in sealing the root canal system in order to prevent recontamination and microbial invasion. However, complete elimination of microorganisms is impossible owing to the anatomical complexity within root canal systems [1,2]. In addition, suboptimal obturation would compromise the 3D seal and might lead to endodontic failure [3,4].

Gutta percha (GP) and root canal sealer are the two main components used to achieve the desirable 3D sealing of the root canal space [5]. An ideal root canal sealer should be capable of creating an effective bond to the GP and root canal walls to prevent micro-leakage at the interface [6,7]. Biocompatibility and bioactivity are essential properties for root canal sealers, as these materials are in close proximation with the surrounding tissues and affect the repair [8,9]. If the sealers are biocompatible and soluble in tissue fluid, minimal extrusions could be tolerated by the peri-radicular tissues [10]. However, this outcome might also slow down or impair the healing process, or, in some cases, induce local inflammation in the periapical region [10,11]. Therefore, the selection of biomaterials is important to avoid risks of postoperative failure for nonsurgical endodontic treatment [11,12].

Epoxy resin-based root canal sealers (RBSs) are considered as the gold standard due to their optimal physicochemical properties [13]. These sealers have widely been used for decades due to their low solubility and disintegration, with adequate dimensional stability [12]. However, Kim et al. [14] reported that resin-based sealers lack bioactive properties or osteogenic potential in comparison to calcium silicate-based sealers (CSBSs). The increase in Ca^2+^ in CSBSs regulates osteoblast proliferation and differentiation [15,16,17,18]. These ions upgrade the expression of bone-associated proteins of osteoblasts [19]. Zayzafoon et al. [20] showed that the increase in extracellular Ca^2+^ causes a considerable inflation of Ca^2+^ concentration within the cell through the calcium channels, activating numerous targets including calcium/calmodulin (CaM)-mediated calcium/calmodulin-dependent protein kinase (CaMK). CaMK2 controls c-fos expression, which is an element of AP-1 transcription factor [21] and ultimately supports osteoblast differentiation. As a result, osteoblasts are induced to mineralise the new bone [22,23]. Therefore, calcium ions in the CSBSs encourage osteoblastic differentiation and bone formation [11]. Lee et al. [11] and Zhang et al. [12] demonstrated a similar phenomenon by showing that Ca^2+^ in the CSBSs stimulates the expression of bone-associated proteins and is required for apatite genesis. Apatites then mediate osteoblastic activity to modulate and mineralise new bone via accumulation of apatite crystals [22]. 

Furthermore, Osiri et al. [24] showed that CSBSs along with the root filling material (Gutta Percha) bonded to the dentine walls and reinforced the prepared root canal system. The authors reported a fracture resistance similar to that of intact roots [24]. In addition, Atteia et al. [10] demonstrated significant improvement in the apical healing and lower dissolution rate with CSBSs when compared to RBSs. Supporting the above observations, Nagar et al. [25] reported that CSBSs showed a superior performance in comparison to RBSs in terms of clinical and radiographic parameters. Contradicting the above evidence, Graunaite et al. [23] stated that a total of 35% of the study population (*n* = 57 patients) was affected with postoperative pain when treated with the CSBSs. There were reported statistically significant differences between the mean values of the VAS scores for the RBS and CSB groups when assessed at 24 h, 48 h, 72 h and seven days post-obturation [23,26,27,28].

Junior et al. [29] and Jamali et al. [30] published systematic reviews comparing the effect of CSBSs and RBS on clinical outcomes, which included five and four controlled randomised clinical trials, respectively. In addition, Mekhdieva et al. [31] evaluated the postoperative pain following warm vertical compaction technique using bioceramic sealer in comparison to cold lateral condensation. However, these reviews conducted previously evaluated only the intensity of post-obturation pain and failed to include parameters such as radiographic healing and absence of clinical symptoms, i.e., sinus healing, reduction in inflammation and absence of tenderness to percussion. In addition, there is still limited evidence in relation to potential clinical and radiographic effects of CSBSs when compared to the RBSs following root canal treatment. Therefore, there is a need for further evidence that combines the clinical and radiographic outcomes and presents consolidated data regarding the effect of calcium silicate-based sealers versus resin-based sealers following non-surgical root canal treatments.

Therefore, this systematic review with meta-analysis was conducted to assess available literature on both the clinical and radiographic outcomes of CSBSs when compared to RBSs following non-surgical endodontic treatment in permanent teeth. 

## 2. Protocol Development

**Figure 1:**
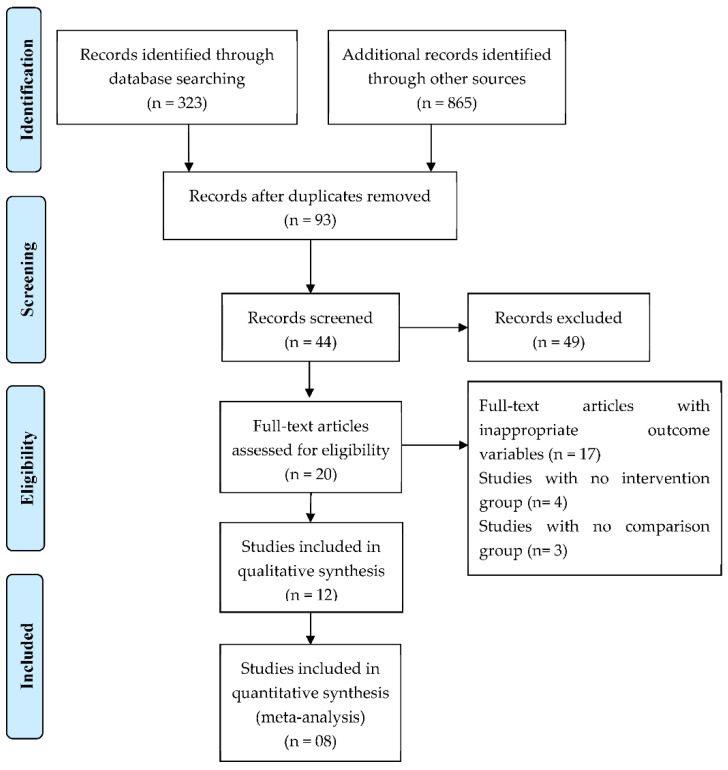
PRISMA flow diagram.

This study was conducted in accordance with the Preferred Reporting Items for Systematic Review and Meta-Analyses (PRISMA) statement (Figure 1) and registered in PROSPERO (CRD42020197475 https://www.crd.york.ac.uk/prospero/record_email.php, accessed on 18 January 2020). The following focused question using PICOs format (Patient, Intervention, Comparison and Outcome) was proposed: “Are calcium silicate-based sealers effective in improving the clinical and radiographic outcomes of endodontically treated permanent teeth in comparison to resin based sealers?”.

### 2.1. Literature Screening and Study Selection

A comprehensive search was conducted in PubMed/MEDLINE, DOAJ, Cochrane Central Register of Controlled Trials and Web of Science to include studies published in all languages (without any limitations) until December 2021. Searches in the ClinicalTrials.gov database and in the references of the included studies (cross-referencing) were also conducted. Google, Greylit and OpenGrey were used to search grey literature. Medical Subject Headings (MeSH) terms, keywords and other free terms related to the PICO question were used with Boolean operators (OR, AND) to combine searches. The same keywords were used for all search platforms following the syntax rules of each database, and the search terms were modified according to the database (Table 1).

### 2.2. Inclusion and Exclusion Criteria Outline According to the PICOs Strategy

The inclusion and exclusion criteria followed is as shown in Table 2.

### 2.3. Screening Process

The search and screening processes were conducted by two authors. Analysation of the titles and abstracts was followed by full-text articles chosen for in-depth reading. The level of agreement, between the two reviewers, calculated by Cohen’s kappa (k), was 0.92 for titles and abstracts and 0.94 for full texts. The third author was approached in case of any differences among reviewers.

### 2.4. Data Extraction

The following data were extracted from the included studies: author names, study design, age of participants, sample size, type of tooth, type of pulpal disease, method of pulp testing, method of root canal preparation, type and concentration of irrigant solution used, obturation technique, endodontic sealers used, medicament prescribed, outcome assessed, method of outcome assessment(s), time of evaluation and authors’ conclusions.

### 2.5. Quality Assessment and Risk of Bias Analysis (ROB)

The quality of the selected studies was assessed using the Cochrane Collaboration Tool [34] for RCTs, including random sequence generation, allocation concealment, blinding of participants, incomplete outcome data, selective reporting and other biases. Methodological index for non-randomised studies (MINORS) was used for quality assessment of the included non-randomised comparative studies [35].

### 2.6. Statistical Analysis

Review Manager (RevMan) 5.3 (Version 5.3, Copenhagen: The Nordic Cochrane Centre, The Cochrane Collaboration, 2014) was used for statistical analysis. The primary outcome was measured as standardised mean difference (SMD) for the mean pain level. However, dichotomous data related to the risk of occurrence of post-obturation pain, the intensity of the pain and frequency of analgesic medicament intake were expressed as relative risks (RRs) at 95% confidence intervals (CIs), *p* < 0.05, using the random-effect model. Heterogeneity was assessed by the Q test, for *p* < 0.1, as well as by the I^2^ test. Sensitivity analysis was conducted to assess the stability of the results.

## 3. Results

The initial electronic database search resulted in 1188 titles. After removal of duplicates and screening of the abstracts, a total of 93 relevant titles were selected by two independent reviewers (VC and AB). Out of these 93 articles, 44 were then chosen for the full-text evaluation, which also included articles through hand searching of the reference lists of the selected studies. Subsequently, a total of 20 studies were selected according to the inclusion and exclusion criteria. 

Seventeen studies with inappropriate outcome variables, four studies with no intervention group, and three studies without comparison group were excluded.

### 3.1. Study Characteristics

The general characteristics of 12 studies [10,23,25,26,27,28,36,37,38,39,40,41] are shown in Table 3. All included studies were unicentric trials published between 2013 and 2021. Geographically, three studies [25,37,41] were performed in India, two in Brazil [26,27] two in Turkey [28,39] and one each in Singapore [36], Russia [38], Lithuania [23], Portugal [40] and Egypt [10]. The study design of nine studies [23,25,26,27,28,36,37,39,40] was RCTs, two studies [38,41] were NRS, and the remaining one [10] was a retrospective study. There was no reported ethical approval in two studies [6,26], whilst three studies failed to mention the informed consent [10,40,41]. A total of 833 permanent anterior or posterior teeth from maxillary and mandibular arches were included in this systematic review. These teeth were diagnosed with irreversible pulpitis, pulp necrosis, or symptomatic or asymptomatic apical periodontitis. The treatment modality was root canal therapy using either CSBSs (n: 445) or RBSs (n: 388).

Between the studies, there were significant methodological heterogeneities observed according to the different position of each tooth (mandible [10,23,27,28,36,39,40,41] or maxilla [23,25,26,27,36,37,40,41]) and tooth type (anterior [23,25,26,27,36,37,40,41] or posterior [10,23,27,28,36,39,40,41]). Both single and multiple visits to complete the root canal treatment were reported. Calcium hydroxide-based dressing was used in studies requiring two or more visits for completion. In five of the included studies, the treatment was performed using dental operating loupes by a specialist or under the supervision of a specialist [23,27,36,38,39]. 

The obturation method varied between single gutta-percha (GP) cone [26,28], vertical compaction using GP [23,27], carrier-based obturation [39] and lateral compaction [10,41]. CSBSs were used in nine studies [10,23,26,27,28,36,38,39,40]; four studies [25,27,28,41] used mineral trioxide aggregate (MTA, Dentsply Tulsa, Johnson City), whilst two studies [25,37] assessed the SmartpasteBio sealer (Endo Technologies, LLC, USA). In addition, AH Plus RBSs was used for comparison in all included studies. CBCT was used alone by five studies to confirm the quality of root canal obturation [10,26,27,36,39].

The primary outcome parameters assessing post-intervention effects varied across studies. Studies measured post-obturation pain variously as mean pain level [23,26,39,41], pain occurrence [23,25,26,27,36,40] and intensity of pain [12,13,22,26,42] from six hours to seven days after the procedure. Visual analogue scale (VAS) was used in seven studies [10,23,26,27,28,39,41], while modified VAS [40] and Likert scale [36] were performed in one study each. Each study assessed post-obturation pain. The need to take analgesic for pain relief [26,28,36,39] and number of tablets consumed for pain relief [26] were also measured after a minimum of 24 h following the root canal therapy.

The secondary outcomes such as extrusion of the sealer [10,26,36], healing of apical lesion [10,25,37,38,41] and resorption of the sealer [10] were assessed using radiovisiography. Clinical assessments were of tenderness on percussion, palpation, presence of sinus tract, swelling and mobility [25]. These were carried out after a minimum of one month following the completion of endodontic therapy. 

A total of twenty studies were included in this review; however, eight studies were then excluded for the meta-analysis. Subsequently, the study by Ved et al. [37] was not considered for further quantitative analysis, as there were only radiographic assessments for the mean area change in the periapical lesion using the Image J software in pixels/mm^2^. Nagar et al. (2018) and Zavattini et al. (2020) analysed clinical (tenderness to percussion assessment, mobility) and radiographic outcomes. However, these authors reported the results in percentages for each group at 1, 3, 6 and 12 months. Therefore, these studies were also excluded from the meta-analysis, since the authors presented the changes in area for healing in percentages only. VAS scale for postoperative pain was also not reported. A study by Atteia et al. (2017) was excluded in the meta-analysis, as this study presented the mean and median values only comparing the effects of extruded CSBS and RBS on apical healing using digital radiography. 

Among the studies that were not included in the quantitative analysis, one study demonstrated optimum healing of the apical lesion following the use of bioceramic sealer in comparison to resin-based sealer after 6 and 12 months post-root canal treatment [37]. In addition, Zavattini et al. [38] demonstrated a high percentage of success rate with the group using the bioceramic sealer in comparison to the resin-based group. However, the authors failed to find statistically significant differences between the two groups. Nagar et al. [25] concluded bioceramic sealer was most efficient in comparison to the MTA, resin-based and zinc oxide eugenol sealers. Similarly, Atteia et al. [10] recorded high observations of 1.67 with Totalfill in comparison to 1.2 with AH Plus sealers (using Mann-Whitney U-test) with respect to complete healing following the RCTs. Using a Student *t*-test, the authors also reported a statistically significant difference (*p* ≤ 0.001) in the digital radiodensity of bioceramics (mean value 37.46), which increased at 12 month recall in comparison to the resin based sealers (mean value 19.73). It was concluded that increased radiodensity and low solubility of bioceramics after 12 months might be indicative of their osteoinductive and osteoconductive potential [10].

### 3.2. Risk of Bias (ROB) and Quality Assessment

**Figure 2:**
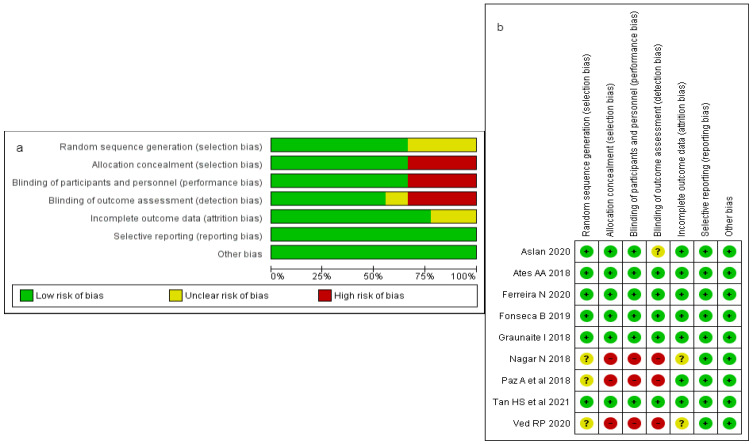
Risk of bias (ROB) and quality assessment. (**a**) Review authors’ judgements about each risk of bias item presented as percentages for the included studies; (**b**) Review authors’ judgements about each risk of bias item for each included study.

Figure 2 shows the quality assessment of the included studies. The main shortcomings were related to allocation concealment, blinding of participants and outcome assessment. The random sequence generation was unclear in three studies [25,37,40], and two studies reported incomplete outcome data [25,37]. MINORS was used for quality assessment of two non-randomised comparative studies [38,41] that presented scores of 21 and 22, respectively, demonstrating low risk of bias (Table 4). This is in accordance with the globally accepted score between 21–24 for non-randomised studies [35].

Financial support was disclosed only by three studies [36,38,39], which might indicate a funding bias.

### 3.3. Quantitative Analysis

A total of eight studies [23,26,27,28,36,39,40,41] fulfilled the inclusion criteria for quantitative analysis. The studies which included more than one type of CSBS group [27,28] or more than one type of obturation techniques as a study group [40] were analysed separately and were considered as different studies.

### 3.4. Mean Pain Levels

**Figure 3:**
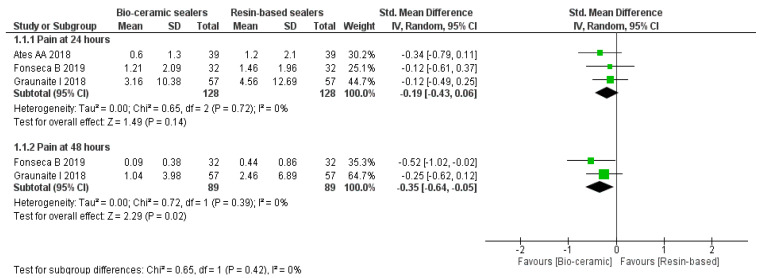
Forest plot demonstrating the comparisons of mean pain levels at 24 and 48 h. between the CSBs and RBSs.

The meta-analysis (Figure 3) was carried out as subgroups analysis using a random-effect model according to the time intervals of 24 and 48 h. At 24 h, there was no significant difference in the mean pain levels (MD, −0.19, 95% CI = −0.43–0.06, *p* = 0.14, I^2^ = 0%). However, significant differences favouring the CSBS group (MD, −0.35, 95% CI = −0.64−0.05, *p* = 0.02, I^2^ = 0%) were observed after 48 h [23,26,39].

### 3.5. Risk of Occurrence of Pain

**Figure 4:**
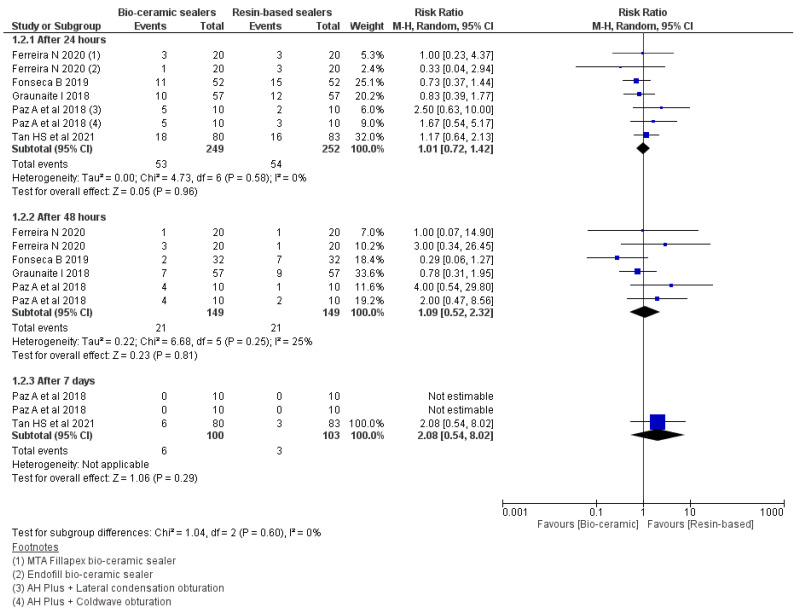
Forest plot showing comparisons of risk of occurrence of pain between CSBSs and RBSs after 24, 48 h and seven days postoperation.

The meta-analysis (Figure 4) was carried out according to the postoperative time intervals of 24, 48 h and seven days between the patients treated with the CSBS and RBS [23,26,27,36,40]. There was no significant difference in the risk of occurrence of pain at 24 (RR: 1.01 95% CI = 0.72–1.42, *p* = 0.96, I^2^ = 0%), 48 h (RR: 1.09 95% CI = 0.52–2.32, *p* = 0.81, I^2^ = 25%) or seven days (RR: 2.08 95% CI = 0.54–8.02, *p* = 0.29).

### 3.6. Intensity of Pain

**Figure 5:**
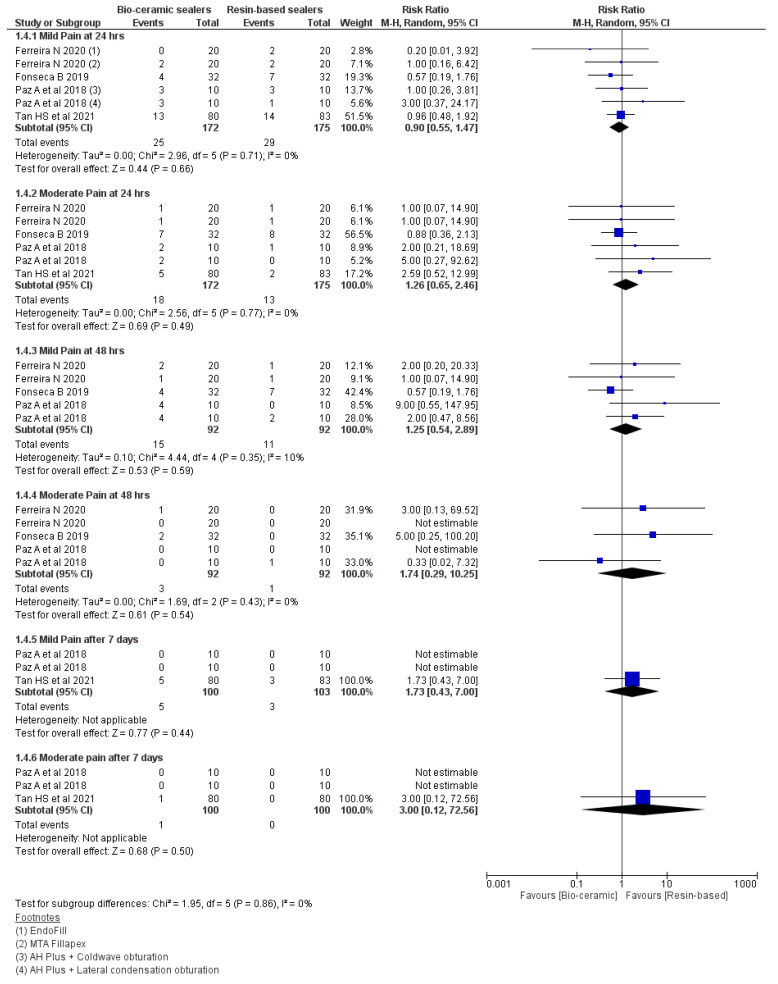
Forest plot demonstrating the comparison of intensity of pain between the CSBSs and RBSs at 24, 48 h and seven days postoperation.

The meta-analysis (Figure 5) failed to demonstrate any differences for the intensity of post-obturation pain for a period of 24 and 48 h., then seven days (Table 5). The severity of pain was measured as mild and moderate [26,27,36,40].

### 3.7. Analgesic Medicament Intake within 24 h

**Figure 6:**
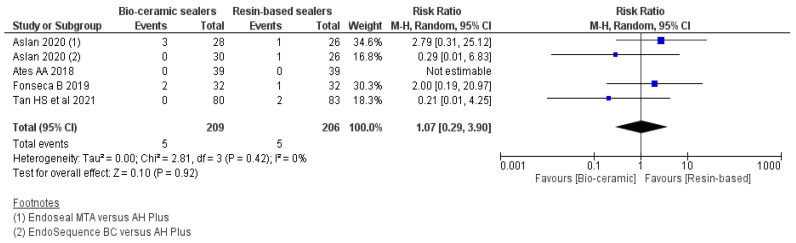
Forest plot showing the comparison of the need for analgesics intake between the CSBSs and RBSs within 24 h.

As per the forest plot (Figure 6), assessing analgesics medicament intake, there were no significant differences in the frequency of analgesics medicament intake within 24 h (RR: 1.07 95% CI = 0.29–3.90, *p* = 0.92, I^2^ = 0%) post-treatment [26,28,36,39].

### 3.8. Extrusion of the Sealer

**Figure 7:**
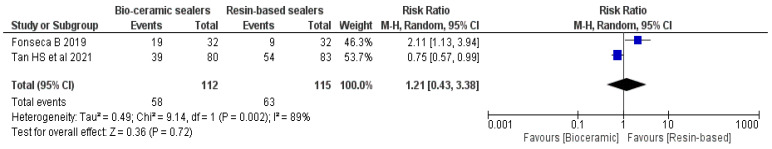
Forest plot comparing the bioceramic and resin-based sealer extrusion.

Meta-analysis reported that both groups failed to show any significant differences (RR: 1.21 95% CI = 0.43-3.38, *p* = 0.72, I^2^ = 89%) in terms of sealer extrusion (Figure 7) [26,36].

## 4. Discussion

Based on the levels of evidence given by the Oxford Centre for Evidence-based Medicine [43], this systematic review and meta-analysis of clinical trials provides level 1 evidence for assessing post-obturation effect of CSBSs as compared to RBSs. In addition, Grading of Recommendations, Assessment, Development and Evaluations (GRADE) was carried out to assess the evidence available for this study. The overall results of the meta-analysis displayed that the CSBSs showed performance superior or similar to the conventional RBS for parameters such as post-obturation pain level, risk of occurrence, intensity of pain at 24 and 48 h and need for analgesic drug intake within 24 h.

Success with non-surgical root canal treatment is attained by the removal of micro-organisms from the canals followed by 3D obturation to prevent reinfection. Clinical and radiographic parameters to evaluate endodontic success include absence of pain, inflammation and other symptoms, absence of sinus tract, retained function and radiological evidence of a normal periodontal ligament space around the root. [44,45]. Sathorn et al. [46] and Wong et al. [47] reported incidence of post-obturation pain between 3 and 58% in patients, with the highest being on the first and second day [36]. This might be due to the composition of the sealer or obturation material. In cases of sealer extrusion, this could cause localised inflammatory response affecting the healing process in the periodontium, which could be related to the possible release of chemical irritants by sealers [12,36]. In addition, many studies suggested that micro-organisms are not completely eliminated during root canal treatment and become the major factor in initiation, development and persistence of apical periodontitis [48,49,50].

The results for the primary outcome, i.e., post-obturation pain, showed no significant difference between the two evaluated sealer groups. However, Graunaite et al. [23] and Fonseca et al. [26] in their respective studies showed that the delayed setting time of AH Plus sealer might affect its biocompatibility and trigger the potential for cytotoxic by-products to be released before the final setting, leading to periapical inflammation that might result in post-obturation pain [23,28]. In addition, Lodienė et al. [51] and Zhang et al. [52] demonstrated significant differences in cytotoxicity levels between the RBS and CSBSs. However, such differences were not observed, as there was no correlation between sealer extrusion and postoperative pain [23,26,28,36,39]. There were statistically no significant differences in the mean pain intensity levels, need for analgesic drug intake and occurrence of post-obturation pain between the CSBS and RBS at any of the assessed time points (24 h. up to seven days). However, the mean pain levels were lower in the CSBSs in comparison to RBSs at 48 h. This could be due to the limited contact of these sealers within periapical tissues [29]. In addition, the presence of tissue fluids might dilute the concentration of toxic substances; therefore, inflammatory response might not be activated [29,33]. 

In all studies except that of Tan et al. [36], the scores for post-obturation pain at 1, 3 and 7 days were recorded using numerical values between 0 and 10 according to the VAS scale and then converted to a verbal scale—“no pain”, “slight pain”, “moderate pain”, and “severe pain”—to assess the intensity of pain. In addition, the number of visits required to complete the root canal treatment and level of complexity of each treatment were not standardised. These factors can cause an outcome reporting bias in the results. Root canal sealers would aim to fill all irregularities within the root canal system if these materials have the desired rate of flow. In this respect, excessive flowability might increase the risk of sealer extrusion [26]. Fonseca et al. [28] stated that the unintentional extrusion of each sealer was recorded and confined to the region immediately adjacent to the portal of the canal exit. Fonseca et al. [26] reported a higher rate of extrusion for the Sealer Plus BC (MKLife Medical and Dental Products, Porto Alegre, Brazil) (59.74%) in comparison to the AH Plus sealer (28.13%). However, this was reversed in the study by Tan et al. [36], where the AH Plus sealer (65%) showed a higher rate of extrusion in comparison to the Sealer Plus BC (48.7%). The meta-analysis reported that there was no significant difference in the extrusion of sealers irrespective of their types. Even though a high rate of extrusion was observed (59.74% Sealer Plus BC; 28.13% AH plus), the VAS using pain perception ranging from 0–10 showed no report of pain by the patients, thus confirming that there is no association between sealer extrusion and post-obturation pain. 

The success rates of these two sealers were measured according to the absence of pain, inflammation, absence of sinus tract, retained function, normal width and continuity of the periodontal ligament space on radiographs, along with the evidence of apical healing, i.e., improved radiodensity. Nagar et al. [25] and Ved et al. [37], using the SmartpasteBio BCS, and Atteia et al. [10], with Totalfill BCS, showed significant improvements in the clinical parameters after 1, 3, 6 and 12 months when compared to the RBS. In addition, complete apical healing, a slower rate of resorption and improved radiodensity assessed radiographically favoured the CSBs group in comparison to the RBS. Increased radiodensity and low solubility rate of the extruded CSBs indicate that these sealers act as osteoinductive and osteoconductive materials that accelerate healing and adsorb more minerals from the surrounding tissue. However, Nagar et al. [25] failed to provide the details of instrumentation and obturation techniques, which could influence the incidence of postoperative pain. These authors also mentioned using pre- and postoperative CBCT scans only for the CSBs group, whilst only digital periapical radiograph was used for the RBS.

The review included 12 clinical studies published between 2013 and 2021. The ages of the participants were above 18 years, with a mix of both genders. The selection bias was minimised by performing sensitivity analysis on quantitative results by excluding the studies with vital teeth [36]. In addition, the participants on any medicines that could possibly interfere with the post-obturation effect of sealers were excluded from this systematic review. However, a few studies failed to mention achieving apical foramina patency [23], which, if achieved, favours the occurrence of unintentional sealer extrusion [26]. Obturation technique for both sealers differs, as these materials were used according to the manufacturers’ instructions. A few included studies [25] failed to mention the cleaning and shaping or obturation technique used.

A Cochrane systematic review by Manfredi et al. [53] concluded that there is lack of evidence suggesting one treatment regimen is better than the other. In this respect, Tan et al. (2021) failed to mention the number of visits required to complete the treatment [36]. In this current review, to rule out the effect of different visits, only single-visit RCT studies were included in the sensitivity analysis. This analysis affirmed the fact that although the exclusion of the studies reduced the RRs and heterogeneity, the overall results remain unaltered.

Furthermore, methodological heterogeneity was noticed due to the location of study, methodology, sample size, number and experience of clinicians performing the procedures and diagnosis, method of root canal preparation and obturation, visits required to complete RCT, marking on the scale used for assessing pain and radiographic techniques. A random-effects model instead of a fixed-effects model for meta-analysis was used to address this heterogeneity. The sensitivity analysis performed using a fixed-effects model for the study outcomes showed unchanged overall results. 

Inter-study variability and inconsistency within studies are identified as limitations in this systematic review. The clinical heterogeneity among the included studies could not be avoided. Individual analyses for tooth types (incisors, canines, premolars and molars), age, gender and number of visits required to complete the treatment were considered in the included studies. In addition, although the studies assessed postoperative pain, the reported data regarding the sealer extrusion and lack of standardisation in the pain relief doses were not comparable to perform the meta-analysis. Only a small number of articles and participants were included for quantitative analysis due to the limited evidence. However, six out of twelve studies demonstrated acceptable methodological validity, exhibiting a low risk of bias [23,26,27,28,36,39,40]. Two studies disclosed the presence of external funding. The funding was received for the sealers used in both studies [36,38]. It could be speculated that the possibility of a funding bias in such cases cannot be overlooked.

Previously published systematic reviews and meta-analysis [29,30,31] evaluated the postoperative pain at different time intervals, with a maximum of only seven days. Junior et al. [29] were unable to perform meta-analysis for sealer extrusion and doses of medications i.e., Ibuprofen. Jamali et al. [30] included articles published in English only, which could lead to selection and selective outcome reporting biases. In addition, Mekhdieva et al. [31] failed to provide a clear description of the inclusion and exclusion criteria used for their study. This current systematic review is the first meta-analysis to assess the effect of CSBSs vs. RBSs on the clinical as well as radiographic outcomes when used for root canal treatment in permanent teeth.

Future randomised clinical trials evaluating postoperative pain and periapical and bone healing with different pulp and periodontal status at varying time intervals at least up to four years are required [54]. In addition, the CONSORT [55] or PRIRATE [56] recommendations need to be followed. Standardising the use of numerical rating scales (0–10 cm) to analyse pain intensity is preferred, as the more levels a tool has, the more sensitive it is, to the point that it could detect even a small change in pain intensity [57].

## 5. Conclusions

The overall results of the present systematic review and meta-analysis demonstrate that the CSBSs presented acceptable performance with similar results to the gold standard RBSs in terms of mean post-obturation pain level, risk of occurrence and intensity of pain at 24 and 48 h, as well as for analgesic drug intake within 24 h and extrusion of sealer. However, the included studies have shortcomings that were presented in this current review. Therefore, further well-designed, controlled randomised clinical trials for a period of at least four years are required to provide high-quality evidence.

## Figures and Tables

**Table 1:** The search strategy and PICOS tool.

Search Strategy	
Focused Question	Are CSBSs sealers effective in improving the clinical and radiographic outcomes of endodontically treated permanent teeth in comparison to RBSs?
Search strategy	
Population (#1)	(Permanent Dentition [MeSH] OR Adult Dentition [Text Word] OR Secondary Dentition [Text Word] OR Permanent teeth [Text Word] OR Teeth [Text Word] OR Root Canal Obturation [MeSH]
Intervention (#2)	(Bioceramic sealer [Text Word] OR Endosequence BC [Text Word] OR iRoot Plus [Text Word] OR MTA Fillapex [Text Word] OR Totalfill BC [Text Word] OR tricalcium phosphate [Text Word] OR tricalcium phosphate ceramic sealer [Text Word] OR Calcium silicate sealer [Text Word] OR Calcium phosphate sealer [Text Word] OR Endodontic sealer [Text Word] OR Root canal sealer [Text Word])
Comparisons (#3)	(Epoxy resin-based root canal sealer [Text Word] OR AH Plus [Text Word] OR Adseal [Text Word] OR AH 26 [Text Word])
Outcomes (#4)	(Success [Text Word] Pain [Text Word] OR Pain intensity [Text Word] OR medication [Text Word] OR duration [Text Word] OR Visual analogue scale [MeSH] OR Heft Parker Visual Analog Scale [Text Word] OR Apical healing [Text Word])
Study design (#5)	(Clinical trials [MeSH] OR randomized controlled studies [Text Word] OR randomized control trials [MeSH] OR randomized control clinical trial MeSH OR non-randomized control trials [Text Word] OR Quasi experimental studies [Text Word] OR before and after study design [Text Word] OR cohort studies [Text Word] OR in vivo study [Text Word])
Search Combination	#1 AND #2 AND #3 AND #4 AND #5
Database search	
Language	No restriction
Electronic Databases	PubMed/MEDLINE, DOAJ, Cochrane Central Register of Controlled Trials, Web of Science
Journals	Journal of Endodontics, International Endodontic Journal, Australian Endodontic Journal, Clinical Oral Investigations, Journal of Conservative Dentistry, Journal of American Dental Association
Period of Publication	1 January 2011 to 31 January 2021

**Table 2:** Inclusion and exclusion criteria.

Inclusion Criteria
Population (P): Studies on patients ≥ 18 years of age requiring non-surgical endodontic treatment on minimum one tooth in mandibular/maxillary region irrespective of gender, race, socio-economic status, or root canal obturation technique were evaluated. Interventions (I): Studies using bioceramics [32,33] as root canal sealers in non-surgical endodontic treatment Comparison (C): Studies using resin-based sealers in root canal treatment. Outcome (O): Studies including either both or each outcome: Primary outcome: Studies assessing mean pain level with occurrence and intensity of the post-obturation pain at a minimum of 24 h follow-up using the numerical scales [visual analogue scale (VAS), Likert scale]. Secondary outcome: Studies assessing frequency of analgesics drug intake by individual’s post-treatment clinical success rate (asymptomatic tooth, sinus tract, tenderness on percussion, swelling, tooth mobility) and periapical status (apical healing, resolution of lesion, sealer resorption, sealer extrusion) post-obturation at a minimum of one month of follow-up using radiovisiography. Study design (S): Clinical trials, RCTs, quasi-experimental studies, non-randomised trials (NRS) and in- vivo studies.
Exclusion criteria
Studies involving patients with a medical history such as uncontrolled diabetes and hypertension or any prolonged chronic systemic illness Studies involving patients taking any analgesic, anti-inflammatory, or antibiotics minimum of seven days before the start of the study. Studies involving patients younger than 18 years of age. Studies involving treatment of vital teeth with reversible pulpitis. Observational study designs, case reports or series, cross-sectional studies and articles that are only reviews. Abstracts without full texts in the database. Studies carried out on animals.

**Table 3:** Characteristics of the included studies.

Study Id	Place of Study	Age of Participants	Sample Size I_1_/I_2_/C	Type of Tooth	Type of Pulpal Disease	Pulp Sensibility Test	Method of Root Canal Preparation	Final Irrigant Used	Obturation Technique I/C	Sealer Used I_1_/I_2_/C	Medicament Prescribed	Visit for RCT	Outcome Assessed	Method of Outcome Assessment	Time of Evaluation	Authors’ Conclusion
Tan, H.S., et al. 2021 [36]	Singapore	21 and above	80/-/83	Maxillary and mandibular anterior and posterior teeth	Vital, non-vital and previously root-filled teeth	-	Nickel– titanium rotary files in crown-down approach	1.25% NaOCl 17% EDTA	Totalfill^®^ BC point/non-standardised GP cones	Totalfill BC/-/AH plus	Ibuprofen if necessary	Single and Multiple	Post-obturation pain	Likert scale	1, 3 and 7 days	There was no significant difference in pain experience between teeth filled using AH Plus or Totalfill BC sealer 1, 3 and 7 days after obturation.
Aslan., T, et al. 2020 [28]	Turkey	18–60	28/30/26	Mandibular first and second molar	Asymptomatic irreversible pulpitis	Thermal and electric pulp test	Nickel–titanium file system Reciproc with a VDW	3 mL of 17% EDTA, 3 mL of 5% NaOCl, 2 mL of distilled water	Single tapered gutta-percha cone	Endoseal MTA/Endosequence BC/AH Plus	Ibuprofen 400 mg only when they encountered severe pain	Single	Pain, frequency of analgesic drug intake	VAS	6, 12, 24 and 48 h and on 3rd, 4th, 5th, 6th and 7th day	Endoseal MTA, Endosequence BC Sealer and AH Plus were not significantly different in terms of the severity of postoperative pain after single-visit root canal treatment.
Ferreira, N., 2020 [27]	Brazil	18 and above	20/20/20	Single rooted anterior teeth and premolars	Pulp necrosis	Cold test Absence of bleeding on access opening	-	5 mL 2.5% NaOCl 5 mL 17% EDTA	Single-cone and vertical compaction technique	EndoFill/MTA Fillapex/ AH Plus	-	Min. 2 visits	Postoperative pain intensity	Level of pain	24 h, 48 h and 7 days	Root canal filling using AH Plus, MTA Fillapex and EndoFill resulted in the same postoperative pain occurrence and intensity and need for analgesic intake.
Ved, R.P., 2020 [37]	India	20–40	10/-/10	Upper central or lateral incisor	Asymptomatic apical periodontitis	-	Rotary Protaper (F3) files	3% NaOCl (2 mL) 17% aqueous EDTA	Syringe method/ cold lateral condensation	Smart seal/-/AH plus sealer	-	Min. 2 visits	Resolution of the lesion	Change in area of the periapical lesion using radiographs	3, 6 and 12 months	Smart seal group showed better healing of the lesion as compared to gutta percha and AH Plus group at both 6 and 12 months following root canal treatment.
Zavattini, A., 2020 [38]	Russia	NR	53/-/51	-	Irreversible pulpitis Necrotic pulp	-	Protaper rotary instruments in a crown-down approach	2% sodium hypochlorite 15% EDTA	Single-cone technique/warm vertical condensation	BioRootTM/-/AH plus	-	Two	Success rate	CBCT images, periapical radiographs	12 months	BioRootTM RCS in combination with single cone resulted in a comparable success rate of cases compared to that of warm vertical condensation and AH plus.
Fonseca, B., 2019 [26]	Brazil	25–55	32/-/32	Single- rooted anterior maxillary teeth	Necrotic pulps	Cold and electric pulp test	VDW Silver motor	17% EDTA 2.5% NaOCl	Single-cone technique	Sealer Plus BC/-/AH Plus	600 mg Ibuprofen every 6 h if they experienced any pain	Single	Postoperative pain intensity	VAS	24, 48, 72 h and 1 week	BG sealer presented significantly more extrusion than RG sealer, which was not associated with pain.
Ates, A.A., 2018 [39]	Turkey	18–65	39/-/39	Mandibular premolar or molar	Devitalised teeth	Electric pulp tester	One Shape system and VDW Silver motor	5 mL 2.5% NaOCl, 5 mL 17% EDTA, and 5 mL sterile saline	Carrier-based obturation system- Hero fill™ Soft-Core obturators	iRoot SP/-/AH Plus	200 mg ibuprofen	Single	Preoperative and postoperative pain rating, frequency of analgesic drug intake	Huskisson 10 cm VAS	6, 12, 24 and 72 h.	iRoot SP sealer was associated with lower analgesic intake than AH Plus sealer.
Graunaite, I., 2018 [23]	Lithuania	35–65	61/-/61	Single-rooted teeth	Asymptomatic apical periodontitis	-	Protaper Gold system driven by an X-Smart endodontic motor	Ultrasonic activation for 30 s with 2.0 mL NaOCl, 2.0 mL 17% EDTA	Warm vertical condensation technique using the Calamus Dual System	Total Fill/-/AH Plus	-	Single	Postoperative pain	VAS	24, 48, 72 h and 7 days	AH Plus and Total Fill perform similarly in terms of the occurrence and intensity of postoperative pain in teeth with AAP with no material extrusion beyond the apex.
Nagar, N., 2018 [25]	India	15–47	16/16/16	Maxillary anterior teeth	Apical periodontitis, small periapical lesion, Root resorption	-	-	2 mL of 2.5% NaOCl and 2 mL of sterile saline followed by 10 mL 17% EDTA	-	Bioceramic sealer/MTA-based sealer/AH Plus	-	-	Pain, tenderness on percussion, sinus tract, swelling and mobility	VAS, radiovisiography measurement scale	1, 3 and 6 months	Bioceramic Sealer was found to be of greatest efficiency followed by MTA, AH PLUS and Zinc Oxide Eugenol for all the evaluated parameters.
Paz, A., et al. 2018 [40]	Portugal	NR	10/10 and 10	Maxillary and mandibular anterior and posterior teeth	Asymptomatic irreversible pulpitis, pulp necrosis or disease that needed retreatment	-	Protaper Next engine driven rotary nickel-titanium files	2.5% NaOCl 10% Citric acid	Single-cone technique -/cold lateral condensation and continuous wave of condensation	BioRoot RCS/AH Plus	Ibuprofen 600 mg if needed	Single and Multiple	Postoperative pain	Modified VAS	24, 48, 72, 96, 120, 144 and 168 h	Single cone + Bioceramic and Continuous wave + resin sealer presented the highest percentage of moderate and the lowest levels of postoperative pain intensity felt, respectively, during the 7 day evaluation period
Atteia, M.H., 2017 [10]	Egypt	20–35	15/-/15	Mandibular first molars	Chronic apical periodontitis	Electronic apex locator	Protaper-NEXT NiTi rotary files	3% NaOCl 2 mL of 17% EDTA	Lateral compaction technique of gutta-percha	Totalfill sealer/-/AH Plus	-	Single	Apical healing, sealer resorption and extruded sealer	Periapical radiographs, digital radiography	12 months	Totalfill recorded higher observations of complete apical healing, compared to AH-Plus.
Thakur, S., 2013 [41]	India	18–50	15/-/15	Single rooted tooth	Apical radiolucency and periapical index Score 2 or more Diagnosis	-	Protaper rotary system	2.5% NaOCl, EDTA and normal saline	Lateral compaction technique	ProRoot MTA/-/AH Plus	-	Multiple	Pain evaluation Periapical status Area measurement	VAS, periapical Index, VixWin Pro digital image analysis software	1 week and 6 months	MTA could be used as a root canal sealer with equal effectiveness compared with epoxy resin- or zinc oxide eugenol-based sealers.

AAP: Asymptomatic apical periodontitis, C: Comparative group, EDTA: Ethylenediaminetetraacetic acid, h: hour, I_1_: Intervention group, I_2_: Intervention group, NaOCl: Sodium Hypochlorite, VAS: Visual analogue scale. NR: Not recorded.

**Table 4:** Methodological index for non-randomized studies (MINORS).

	Clearly Stated Aim	Inclusion of Consecutive Patients	Prospective Collection of Data	Endpoints Appropriate to the Aim of the Study	Unbiased Assessment of the Study Endpoint	Follow-Up Period Appropriate to the Aim of the Study	Loss to Follow-Up Less than 5%	Prospective Calculation of the Study Size	* An Adequate Control Group	* Contemporary Groups	* Baseline Equivalence of Groups	* Adequate Statistical Analyses	Total
Thakur et al., 2013	2	1	2	2	2	2	2	0	2	2	2	2	21
Zavattini et al., 2020	2	2	2	2	2	2	0	2	2	2	2	2	22

The items are scored 0 (not reported), 1 (reported but inadequate) or 2 (reported and adequate). The global ideal score is 16 for non-comparative studies and 24 for comparative studies. * For study with control group.

**Table 5:** Observations for the intensity of pain after 24, 48 h and seven days.

Pain Intervals	Observations
24 h	Mild (RR: 0.90 95% CI = 0.55–1.47, *p* = 0.66, I^2^ = 0%) and
	Moderate (RR: 1.26 95% CI = 0.65–2.46, *p* = 0.49, I^2^ = 0%)
48 h	Mild (RR: 1.25 95% CI = 0.54–2.89, *p* = 0.59, I^2^ = 10%)
	Moderate (RR: 1.74 95% CI = 0.29–10.25, *p* = 0.54, I^2^ = 0%)
Seven days	Mild (RR: 1.73 95% CI = 0.43–7.00, *p* = 0.44)
	Moderate (RR: 3.00 95% CI = 0.12–72.56, *p* = 0.50)

## Data Availability

The data presented in this study are available in the manuscript itself.
